# Distinct Energy-Coupling Factor Transporter Subunits Enable Flavin Acquisition and Extracytosolic Trafficking for Extracellular Electron Transfer in Listeria monocytogenes

**DOI:** 10.1128/mbio.03085-22

**Published:** 2023-02-06

**Authors:** Rafael Rivera-Lugo, Shuo Huang, Frank Lee, Raphaël Méheust, Anthony T. Iavarone, Ashley M. Sidebottom, Eric Oldfield, Daniel A. Portnoy, Samuel H. Light

**Affiliations:** a Department of Molecular and Cell Biology, University of California, Berkeley, Berkeley, California, USA; b Duchossois Family Institute, University of Chicago, Chicago, Illinois, USA; c Department of Microbiology, University of Chicago, Chicago, Illinois, USA; d Department of Plant and Microbial Biology, University of California, Berkeley, Berkeley, California, USA; e Génomique Métabolique, CEA, Genoscope, Institut François Jacob, Université d’Évry, Université Paris-Saclay, CNRS, Evry, France; f QB3/Chemistry Mass Spectrometry Facility, University of California, Berkeley, Berkeley, California, USA; g Department of Chemistry, University of Illinois at Urbana-Champaign, Urbana, Illinois, USA; Universite de Geneve

**Keywords:** cofactor trafficking, energy-coupling factor transporters, extracellular electron transfer

## Abstract

A variety of electron transfer mechanisms link bacterial cytosolic electron pools with functionally diverse redox activities in the cell envelope and extracellular space. In Listeria monocytogenes, the ApbE-like enzyme FmnB catalyzes extracytosolic protein flavinylation, covalently linking a flavin cofactor to proteins that transfer electrons to extracellular acceptors. L. monocytogenes uses an energy-coupling factor (ECF) transporter complex that contains distinct substrate-binding, transmembrane, ATPase A, and ATPase A′ subunits (RibU, EcfT, EcfA, and EcfA′) to import environmental flavins, but the basis of extracytosolic flavin trafficking for FmnB flavinylation remains poorly defined. In this study, we show that the EetB and FmnA proteins are related to ECF transporter substrate-binding and transmembrane subunits, respectively, and are essential for exporting flavins from the cytosol for flavinylation. Comparisons of the flavin import versus export capabilities of L. monocytogenes strains lacking different ECF transporter subunits demonstrate a strict directionality of substrate-binding subunit transport but partial functional redundancy of transmembrane and ATPase subunits. Based on these results, we propose that ECF transporter complexes with different subunit compositions execute directional flavin import/export through a broadly conserved mechanism. Finally, we present genomic context analyses that show that related ECF exporter genes are distributed across members of the phylum *Firmicutes* and frequently colocalize with genes encoding flavinylated extracytosolic proteins. These findings clarify the basis of ECF transporter export and extracytosolic flavin cofactor trafficking in Firmicutes.

## INTRODUCTION

Flavins are an essential family of redox-active cofactors that catalyze electron transfer in diverse enzymes (1). Flavin mononucleotide (FMN) and flavin adenine dinucleotide (FAD) are synthesized from the precursor riboflavin (also known as vitamin B_2_). FMN and FAD are the most common types of protein-bound flavins and are nearly ubiquitous throughout the three domains of life. While the role of flavin-binding proteins (flavoproteins) in cytosolic redox activities is well established, the importance of flavins for extracytosolic activities in prokaryotic biology has become increasingly apparent (2–4).

**FIG 1:**
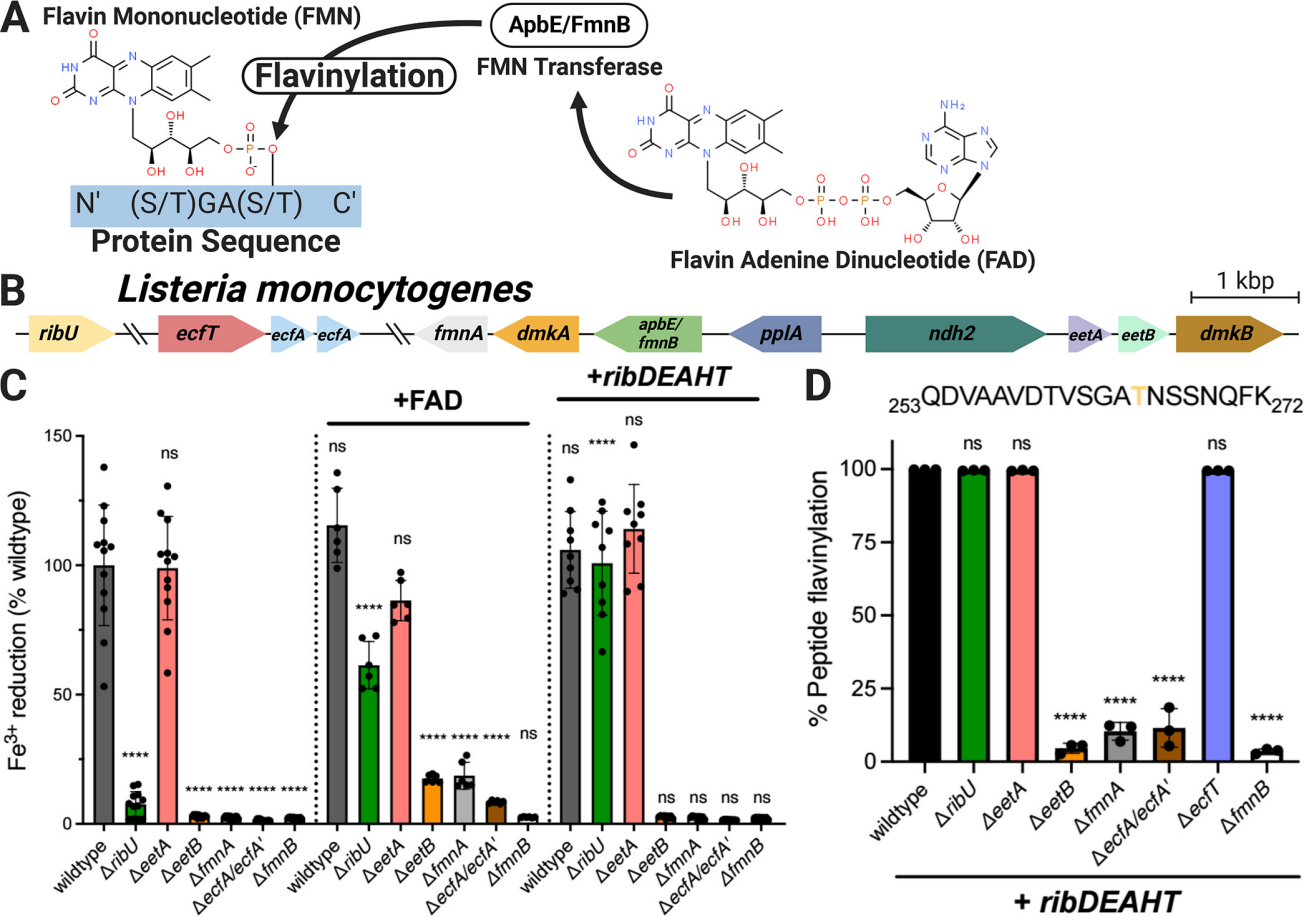
ECF transporter subunits and *eetB* are essential for provisioning flavins to the extracytosolic ApbE homolog FmnB in L. monocytogenes. (A) Reaction catalyzed by AbpE/FmnB flavin transferases. (B) Genomic organization of L. monocytogenes genes addressed in this study. (C) Ferric iron reductase activity of L. monocytogenes strains grown in chemically defined medium. The dotted lines separate flavin auxotroph (wild-type) strains (*n *= 4), flavin auxotroph strains supplemented with extracellular FAD (*n *= 2), and flavin prototroph (+*ribDEAHT*) strains (*n *= 3). For the noncomplemented strains, statistical significance was determined by one-way analysis of variance (ANOVA) and Dunnett’s posttest using the wild type as the control. For the FAD- and *ribDEAHT*-complemented conditions, statistical significance was determined by performing a *t* test compared to the parental noncomplemented condition. (D) Flavinylation levels (flavinylated peptide ion count/total peptide ion count) of the indicated PplA tryptic peptides in flavin prototroph (+*ribDEAHT*) L. monocytogenes strains as assessed by MS. The data are shown as the means and standard deviations (SDs) from three independent experiments. Statistical significance was determined by one-way ANOVA and Dunnett’s posttest using the wild type as the control. ****, *P* < 0.0001; ns, not significant (*P* > 0.05).

In prokaryotes, many extracytosolic flavoproteins are posttranslationally linked to their flavin cofactors (flavinylated) through the action of the ApbE enzyme (5). ApbE specifically uses FAD as a substrate, catalyzing a reaction that links the FMN moiety of the molecule to a serine/threonine residue in substrate proteins via a phosphodiester bond (Fig. 1A) (6). Approximately 50% of sequenced bacterial genomes encode proteins flavinylated by ApbE, with ApbE substrates having been implicated in a wide array of redox-dependent activities (4). For example, *Rhodobacter* nitrogen fixation (Rnf) and NADH:quinone oxidoreductase (Nqr) are prominent multisubunit complexes with ApbE-flavinylated subunits that possess important roles in diverse bacteria and energy metabolisms (7, 8).

Extracellular electron transfer (EET) describes a class of microbial activities that result in the transfer of electrons from the cytosol to the outside of the cell and often function in anaerobic respiration. We previously found that the foodborne pathogen Listeria monocytogenes possesses EET activity that enhances anaerobic growth (9, 10). We further identified an eight-gene cluster responsible for EET (Fig. 1B) (11). Within this cluster, we found that the *fmnB* gene encoded an ApbE-like protein that flavinylated a second protein from the cluster, PplA, at two sites (11, 12). Homologous genes are present in a number of related members of the *Firmicutes* and have been implicated in similar EET activities in several bacteria (13–17). We further found that FmnB is essential for the flavinylation of the extracytosolic fumarate reductase FrdA, a flavin reductase encoded outside the EET gene cluster that uses fumarate as an electron acceptor (18). Strikingly, numerous extracytosolic flavin reductases that are related to fumarate reductase but use different electron acceptors (such as the small molecule urocanate) are found in diverse bacteria (4, 18, 19). Proteins flavinylated by FmnB in L. monocytogenes and related bacteria thus play critical roles in electron transfer to a variety of different extracytosolic electron acceptors.

Extracytosolic proteins acquire cofactors through distinct mechanisms. Some proteins are loaded with their cofactors in the cytosol and then transported by the twin-arginine translocation secretion system across the cytoplasmic membrane in a folded state (20). Other proteins are transported across the cytoplasmic membrane in an unfolded state by the Sec secretion system and fold into their active conformation in the extracytosolic space (20). The latter scenario requires an extracytosolic supply of the cofactor, which can be accomplished by transport from the cytosol. For example, the CcsBA transporter transfers heme across the cytoplasmic membrane and is required for loading heme cofactors into cytochromes (21).

The extracytosolic localization of FmnB and other ApbEs necessitates a source of extracytosolic FAD. We previously proposed that L. monocytogenes FAD was supplied by an energy-coupling factor (ECF) transporter that contained FmnA and RibU subunits (11). These functional assignments were made because FmnA and RibU exhibit high sequence homology to characterized flavin transporter subunits and because PplA is unflavinylated in Δ*fmnA* and Δ*ribU* strains but is rescued by the application of exogenous FAD (11, 22, 23).

ECF transporters are a widespread class of bacterial transporters that have been implicated in the import of many metabolites (24). ECF transporters are generally comprised of four protein subunits, which include a pair of ATPases (ECF-A and ECF-A′), a transmembrane domain (ECF-T), and a substrate-binding subunit (ECF-S). While ECF-S subunits that transport different substrates exhibit highly variable sequences, structural studies have revealed a conserved ECF-S fold. ECF transporters have previously been identified in bacterial genomes on the basis of (i) gene colocalization (ECF transporter subunits often cluster in genomes) and (ii) the high sequence homology of ECF-T, ECF-A, and ECF-A′ subunits (24).

Here, we reevaluate the basis of FAD secretion in L. monocytogenes. We show that RibU is dispensable for FAD secretion under conditions where it is not required for flavin uptake and identify a second protein in the EET gene cluster, EetB, that serves as the ECF-S subunit for FAD export. We identify homologous genes in many bacterial genomes and find that they often colocalize with *apbE*, suggesting a conserved role in flavinylation. These studies reveal the complex basis of ECF transporter function in flavin acquisition and trafficking.

## RESULTS

### RibU is dispensable for PplA flavinylation when flavin auxotrophy is relieved.

Based on the observation that the L. monocytogenes Δ*ribU* strain exhibited diminished EET activity and PplA flavinylation, we previously proposed that RibU served as the ECF-S subunit of a putative FAD transporter responsible for the export of FAD from the cytosol to extracytosolic FmnB (11). However, we subsequently discovered that RibU transports multiple flavins into the cell, and this led us to consider an alternative explanation for the observed phenotypes (25). Because L. monocytogenes is a flavin auxotroph, impaired flavin import likely leads to a diminished cytosolic flavin level in the Δ*ribU* strain, and this could indirectly impair FAD secretion (25). To address this possibility, we employed a previously described prototrophic L. monocytogenes strain that is relieved of flavin auxotrophy through the heterologous expression of the Bacillus subtilis riboflavin *ribDEAHT* biosynthesis operon (25). Consistent with RibU being important for maintaining cytosolic flavin levels in wild-type L. monocytogenes, the expression of *ribDEAHT* in the Δ*ribU* strain restored EET activity and PplA flavinylation to wild-type levels (Fig. 1C and D). In contrast, *ribDEAHT* expression in the Δ*fmnB* strain, which lacks the flavin transferase and thus is essential for PplA flavinylation regardless of cytosolic flavin levels, failed to rescue the defect in EET (Fig. 1C). These results thus provide evidence that decreased cytosolic flavin concentrations account for the observed Δ*ribU* phenotype and demonstrate that RibU is dispensable for trafficking extracytosolic FAD required for EET.

### FmnA and EetB are required for PplA flavinylation in the absence of exogenous FAD.

Having ruled out RibU as a subunit of the FAD exporter, we sought to determine alternative genes essential for FAD secretion. We previously identified *fmnA* as encoding a protein on the EET locus with homology to an ECF-T subunit and found that it was essential for the EET activity of cells grown in rich media but that this phenotype could be reversed by the application of exogenous FAD (11). We thus asked how the Δ*fmnA* strain responded to engineered flavin prototrophy. In contrast to the Δ*ribU* strain, we found that the expression of *ribDEAHT* had no effect on the EET activity and PplA flavinylation of the Δ*fmnA* strain (Fig. 1C and D). This result supported our original interpretation of FmnA representing an ECF-T transporter subunit and suggested that its corresponding ECF-S subunit had been previously overlooked.

We next turned to the question of the identity of the ECF-S that acts with FmnA in FAD export. Since the ECF-S subunit of the FAD exporter should be essential for EET activity, we reasoned that the ECF-S gene was likely localized to the EET gene cluster. As *eetA* and *eetB* provided the only genes without an assigned function and encode membrane proteins consistent with a transporter function, we reasoned that they presented the strongest candidates. We generated Δ*eetA* and Δ*eetB* strains and tested their EET activity. In contrast to the previously described *eetA* transposon mutant, the EET activity of the Δ*eetA* strain did not differ from that of the wild type, suggesting that the previously observed phenotype may have been due to a polar effect caused by transposon insertion (11). In contrast, the Δ*eetB* strain had negligible EET activity and thus remained a promising ECF-S candidate (Fig. 1C).

We next assessed the flavinylation status of PplA in Δ*eetB* strains. Proteomic analysis of L. monocytogenes cells revealed that PplA was unflavinylated in the Δ*eetB* strain (Fig. 1D). Consistent with the lack of PplA flavinylation and impaired EET activity resulting from compromised FAD trafficking, supplementation of Δ*eetB* cells with exogenous FAD partially restored the EET activity (Fig. 1C). Importantly, the lack of PplA flavinylation and EET activity of the Δ*eetB* strain was maintained in the RibDEAHT flavin prototroph background, ruling out diminished cytosolic flavin concentrations as the source of the Δ*eetB* phenotype (Fig. 1C). These findings demonstrate a role for EetB in FAD export.

### EetB is an FAD-binding protein that resembles characterized ECF-S subunits.

**FIG 2:**
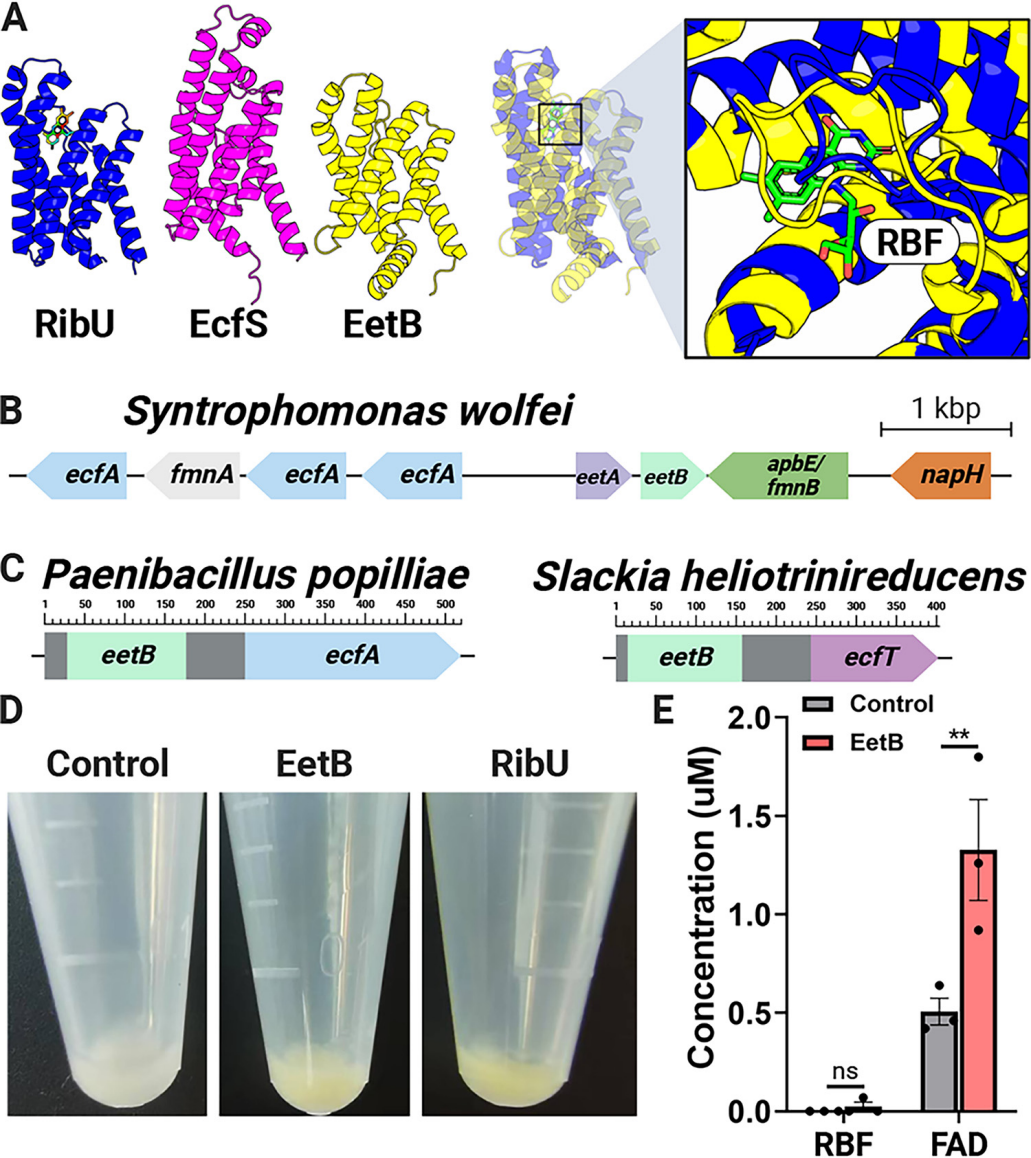
EetB resembles ECF substrate-binding proteins and binds FAD. (A) Comparison of the AlphaFold model of EetB to crystal structures of ECF riboflavin and pantothenate substrate-binding subunits, RibU (PDB accession number 5KBW) and EcfS (PDB accession number 4RFS), respectively. Riboflavin (RBF) is represented as a stick in the RibU structure. (B) Representative bacterial gene cluster in which *eetB* neighbors genes with ECF-T, ECF-A, and ECF-A′ domains. (C) Domain architecture of representative bacterial proteins (NCBI accession numbers WP_006284433.1 and WP_012799238.1) that contain *eetB* and ECF domains. (D) Induced E. coli cell pellets containing *ribU*, *eetB*, and plasmid control overexpression vectors. (E) Riboflavin and FAD pulled down from E. coli cells containing *eetB* and plasmid control overexpression vectors. The means and standard errors of the means (SEMs) from three independent experiments are shown. Statistical significance was determined by performing a *t* test comparing the plasmid control and *eetB* overexpression vectors. **, *P* < 0.01; ns, not significant (*P* > 0.05).

As our genetic studies supported the conclusion that *eetB* is essential for FAD secretion, we questioned whether EetB was the FAD-exporting ECF-S. Since experimentally characterized ECF-S subunits with distinct substrate specificities possess low sequence homology but similar tertiary structures, we used AlphaFold structure modeling software to interrogate the structural attributes of EetB (26–28). The predicted AlphaFold model of EetB revealed striking structural similarity to RibU (root mean square deviation of 4.1 Å) and other experimentally characterized ECF-S subunits, bolstering the case for the protein possessing ECF-S functionality (Fig. 2A).

Genes encoding subunits of characterized ECF transporters are often contained in an operon. We thus reasoned that the genomic context of *eetB* genes could provide additional evidence of ECF functionality. Indeed, we identified *eetB* genes in several bacterial genomes that colocalized with genes encoding ECF-T and ECF-A/ECF-A′ subunits (Fig. 2B; see also Data Set S1 in the supplemental material). Following a similar logic regarding the relationship of functionally related proteins, protein subunits that form a multiprotein complex in one organism are often contained in a single polypeptide chain within other organisms (29). The observation of multiple genes from different bacterial genomes that encode proteins with EetB and ECF-T domains (e.g., NCBI accession numbers WP_021725833.1, MBE6480012.1, and MBQ3267887.1) or EetB and ECF-A domains (e.g., NCBI accession numbers WP_006284433.1, WP_111154897.1, and WP_143797423.1) thus provides additional support for the attributed role of EetB as an ECF-S (Fig. 2C).

10.1128/mbio.03085-22.1 DATA SET S1 Genomic context of *eetB* genes in representative GTDB genomes. Download Data Set S1, XLSX file, 4.4 MB. Copyright © 2023 Rivera-Lugo et al. 2023 Rivera-Lugo et al. https://creativecommons.org/licenses/by/4.0/ This content is distributed under the terms of the Creative Commons Attribution 4.0 International license.

To address whether EetB might be a flavin-transporting ECF-S similar to RibU, we expressed *ribU* and *eetB* in Escherichia coli. We observed that both proteins localized to the membrane-containing insoluble fraction of the resulting cell lysates and presented a yellowish hue, consistent with an association with flavins (which are naturally yellow) (Fig. 2D). To address the hypothesized flavin-binding activity of EetB, we measured flavin levels in *eetB*-overexpressing E. coli cells and found that EetB pulled down FAD (Fig. 2E). Collectively, these analyses demonstrate that EetB is an FAD-binding protein that possesses an ECF-S-like structure consistent with a transport function.

### ECF ATPases are required for PplA flavinylation in the absence of exogenous FAD.

Previous ECF transporter characterizations focused on small-molecule import. As the putative EetB-FmnA complex is the first transporter identified with apparent export activity, we sought to clarify its mechanism of action. ECF importers typically require two ATPase subunits (ECF-A and ECF-A′) for transporter function. As some bacteria use the same ATPase subunits to engage multiple ECF transport systems, we reasoned that the ATPases that function in the RibU ECF transporter flavin import system might also participate in FAD secretion (30, 31). To test this hypothesis, we generated a Δ*ecfA*/Δ*ecfA*′ strain that lacked both of the previously characterized RibU ATPases (19). Consistent with EcfA/EcfA′ being essential for FAD export, the Δ*ecfA*/Δ*ecfA*′ strain was deficient in EET activity and PplA flavinylation and resembled Δ*fmnA* and Δ*eetB* strains in its response to *ribDEAHT* expression and exogenous FAD application (Fig. 1C). These findings thus provide evidence that EcfA/EcfA′ provide dual functions in flavin import and export.

### Structural models illuminate ECF transporters with distinct subunit compositions.

**FIG 3:**
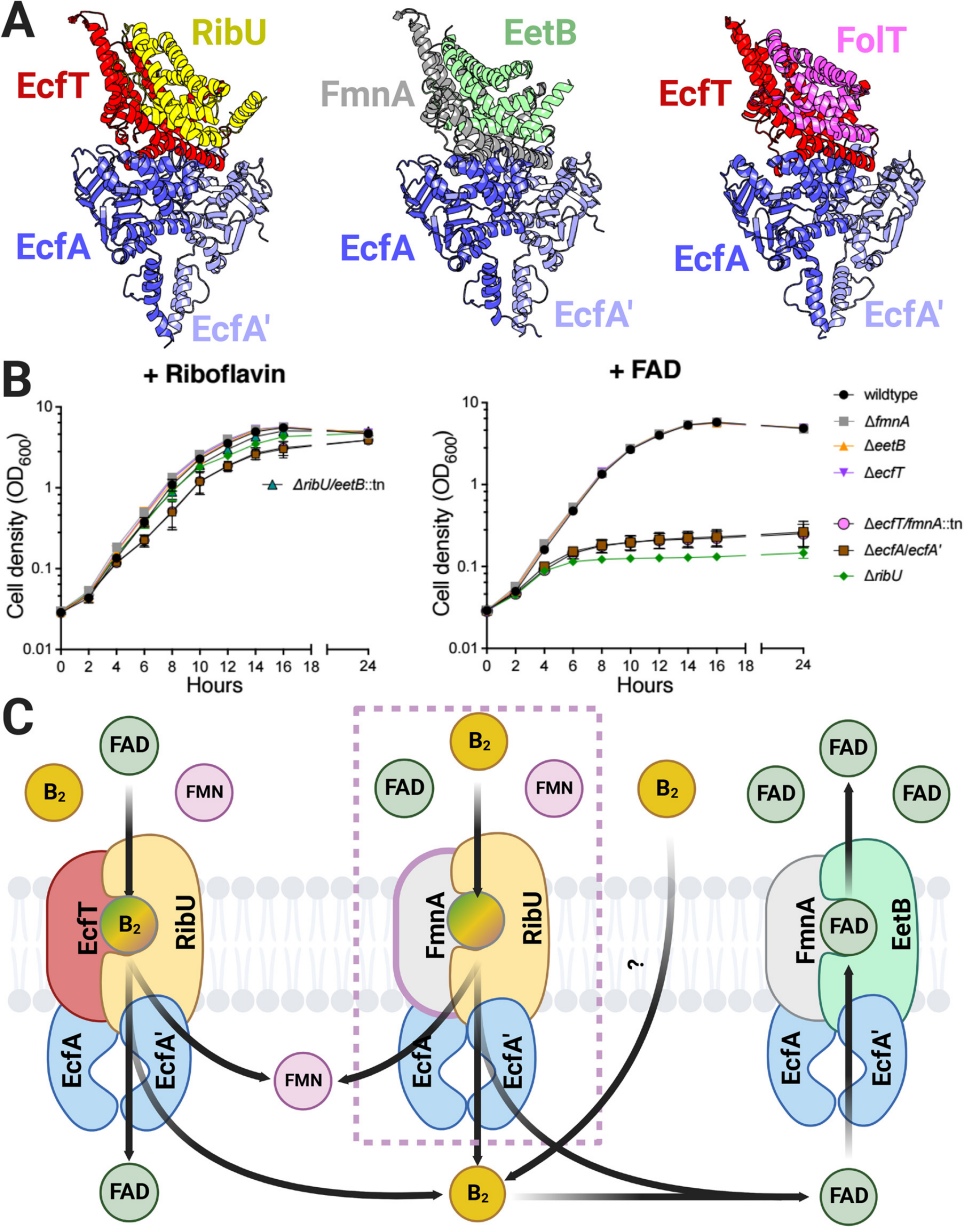
ECF transporter subunits are partially functionally redundant for flavin import and export in L. monocytogenes. (A) Comparison of AlphaFold-multimer RibU-EcfT-EcfA-EcfA′ and EetB-FmnA-EcfA-EcfA′ complex models to a crystal structure of the FolT-EcftT-EcfA-EcfA′ folate ECF transporter (PDB accession number 4HUQ). (B) Growth curves of the indicated L. monocytogenes strains grown in chemically defined medium supplemented with riboflavin or FAD as the sole flavin source. Means and SDs from three independent experiments are shown. (C) Model of ECF complexes that function in L. monocytogenes flavin import and export based on the observed phenotypes.

Previous studies suggested that RibU, EcfT, EcfA, and EcfA′ form an ECF transporter responsible for riboflavin, FMN, and FAD import in L. monocytogenes (23, 25, 32). In contrast, the phenotypes of the L. monocytogenes Δ*eetB*, Δ*fmnA*, and Δ*ecfA*/Δ*ecfA*′ strains identified in our studies suggest that FAD export occurs through an ECF transporter with EetB, FmnA, EcfA, and EcfA′ subunits (Fig. 1C). To further address the feasibility of the distinct implied modes of flavin transport, we used AlphaFold-multimer software to model the putative RibU/EcfT/EcfA/EcfA′ and EetB/FmnA/EcfA/EcfA′ complex structures. Both resulting transporter structural models exhibit striking similarity to a previously determined crystal structure of the folate ECF transporter (Fig. 3A). These structural models are thus broadly consistent with the idea that ECF transporters with distinct subunit compositions could be responsible for the observed phenotypes.

### EcfT and FmnA ECF-T subunits are functionally redundant in flavin import.

We next sought to address how the direction of transport (import versus export) could be achieved through related ECF transporters. L. monocytogenes is a flavin auxotroph, and we previously found that *ribU* was essential for growth under conditions where FMN or FAD is the sole available flavin (25). In contrast to these flavin nucleotides, the Δ*ribU* strain lacked a phenotype in the presence of riboflavin (25). While it thus seemed plausible that EetB could contribute to riboflavin uptake, we found that a Δ*ribU*/Δ*eetB*::Tn strain grew similarly to wild-type L. monocytogenes in chemically defined medium that contained riboflavin as the sole flavin (Fig. 3B). These results suggest that EetB does not substitute for the RibU ECF-S in flavin uptake and that riboflavin can be imported through a presently unknown alternative mechanism.

We next asked about the functional redundancy of ECF-T subunits. While the observation that FmnA is essential for PplA flavinylation suggests that EcfT cannot substitute in FAD export, we wondered if the converse was true (i.e., whether FmnA could facilitate flavin import in the absence of EcfT). Since RibU was essential for growth when FAD was the sole flavin present, we tested ECF-T mutants under this condition (25). The growth of the Δ*ecfT* and Δ*fmnA* strains resembled that of wild-type L. monocytogenes under conditions with riboflavin or FAD (Fig. 3B). In contrast, the Δ*ecfT*/Δ*fmnA*::Tn strain failed to grow in the presence of FAD, similar to the Δ*ribU* and Δ*ecfA*/Δ*ecfA*′ strains (Fig. 3B). These results suggest that both EcfT and FmnA provide functionally viable ECF-Ts for RibU-mediated flavin import and underscore the flexible ECF-T subunit usage for flavin import (Fig. 3C).

### Gene colocalization suggests a conserved role for EetB in ApbE-associated flavin trafficking.

**FIG 4:**
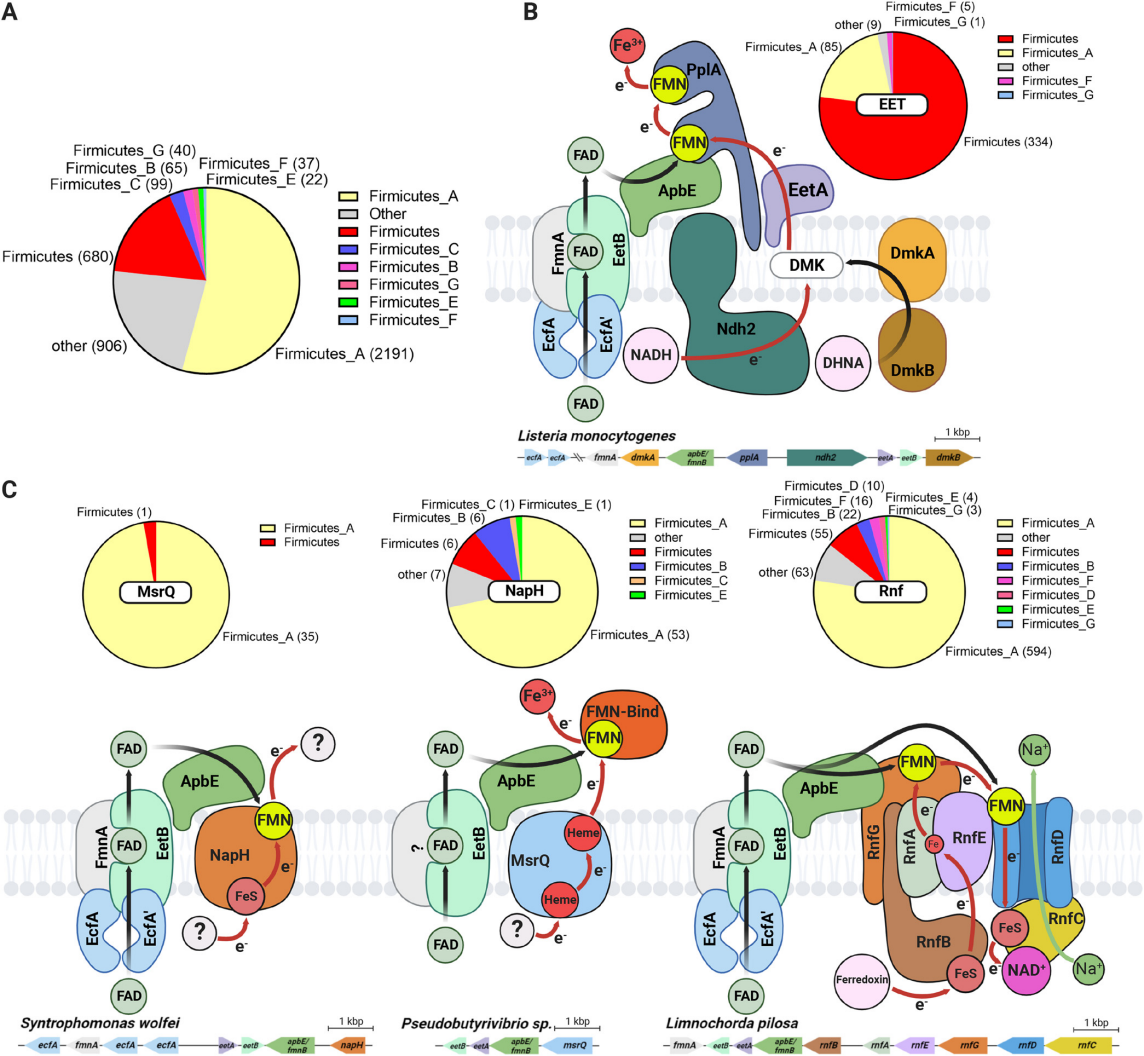
*eetB* genes frequently colocalize with ApbE-associated extracytosolic flavinylation system genes in bacterial genomes. (A) Pie chart showing the number of GTDB reference genomes/phylum that contain an *eetB* homolog (see Data Set S1 in the supplemental material for additional context). (B) Proposed basis of flavin export for L. monocytogenes flavinylation and EET activity. DHNA and DMK refer to 1,4-dihydroxy-2- naphthoate and demethylmenaquinone, respectively. The pie chart shows the number of GTDB reference genomes that contain a gene cluster with both EET and *eetB* genes. (C) Proposed models of flavin export in previously identified flavinylation-associated MsrQ-like, NapH, and Rnf complexes and speculative electron transfer pathways (4). The pie charts show the number of GTDB reference genomes that contain a system gene cluster with an *eetB* homolog.

Having established EetB-FmnA function in FAD export, we next sought to address whether this transporter might be important for FAD trafficking in other microbes. We reasoned that the unique substrate-binding subunit provided the clearest marker of likely FAD export activity, and thus, we searched for *eetB* homologs (Pfam family PF07456) within a collection of 31,910 genomes representative of the genetic diversity of prokaryotes (33, 34). We identified 4,214 bacterial genomes that contain an *eetB* homolog. Taxonomic analyses revealed that *eetB* homologs are most abundant in *Firmicutes* but are also present in a subset of genomes from other phyla (Fig. 4A; Data Set S1).

To clarify the role of EetB in diverse microbes, we next applied a guilt-by-association-based analysis. Our approach took advantage of the frequent colocalization of genes with related functions on prokaryotic genomes and was previously employed to differentiate distinct subtypes of ApbE flavinylation based on colocalization with *apbE* (4). Inspecting the genes that colocalized with *eetB* genes led to several insights, which are elaborated on below.

Supporting the proposed relationship of EetB with other ECF transporter subunits, we found that *eetB* colocalized with ECF-T subunit genes in 291 genomes and with ECF-A/ECF-A′ subunits in 796 genomes, often with ECF subunit genes arranged in an apparent operon. Additionally, *eetB* colocalized with *eetA* in 3,328 genomes. While the L. monocytogenes Δ*eetA* strain lacked an EET phenotype, this conserved synteny suggests a functional link between EetA and EetB. Further supporting this functional association, we identified genes encoding a single polypeptide chain with both EetA and EetB domains in 14 genomes (Data Set S1).

We also found that *eetB* colocalizes with *apbE* on 2,169 genomes. ApbE proteins have been shown to function in multiple extracytosolic redox activities, and we recently proposed operational definitions that enable the identification of gene clusters encoding 10 types of ApbE-flavinylated systems with different mechanisms of membrane electron transfer or substrate specificity (4). We applied these definitions to determine which types of ApbE-flavinylated systems colocalize with *eetB* and found that *eetB* most commonly colocalizes with Rnf and EET systems but also some MsrQ-like and NapH-like systems (Fig. 4B to E; Data Set S1). The association with Rnf was particularly pronounced in the class Clostridia, with *eetB* being colocalized with Rnf genes in 572 genomes. These results thus suggest that ECF exporters traffic FAD to a functionally diverse subset of bacterial ApbE proteins.

## DISCUSSION

The studies presented here establish the basis of FAD trafficking in L. monocytogenes and, notably, provide the most extensive evidence of the role of ECF-like transporters in small-molecule export. Strikingly, our comparison of strains deficient in various ECF components supports the existence of distinct import and export transporters that share subunits (ECF-A/ECF-A′) and exhibit partial functional redundancies (import ECF-T subunits). These findings thus suggest a complex basis of bidirectional flavin transport across the L. monocytogenes cytoplasmic membrane (Fig. 3C).

**FIG 5:**
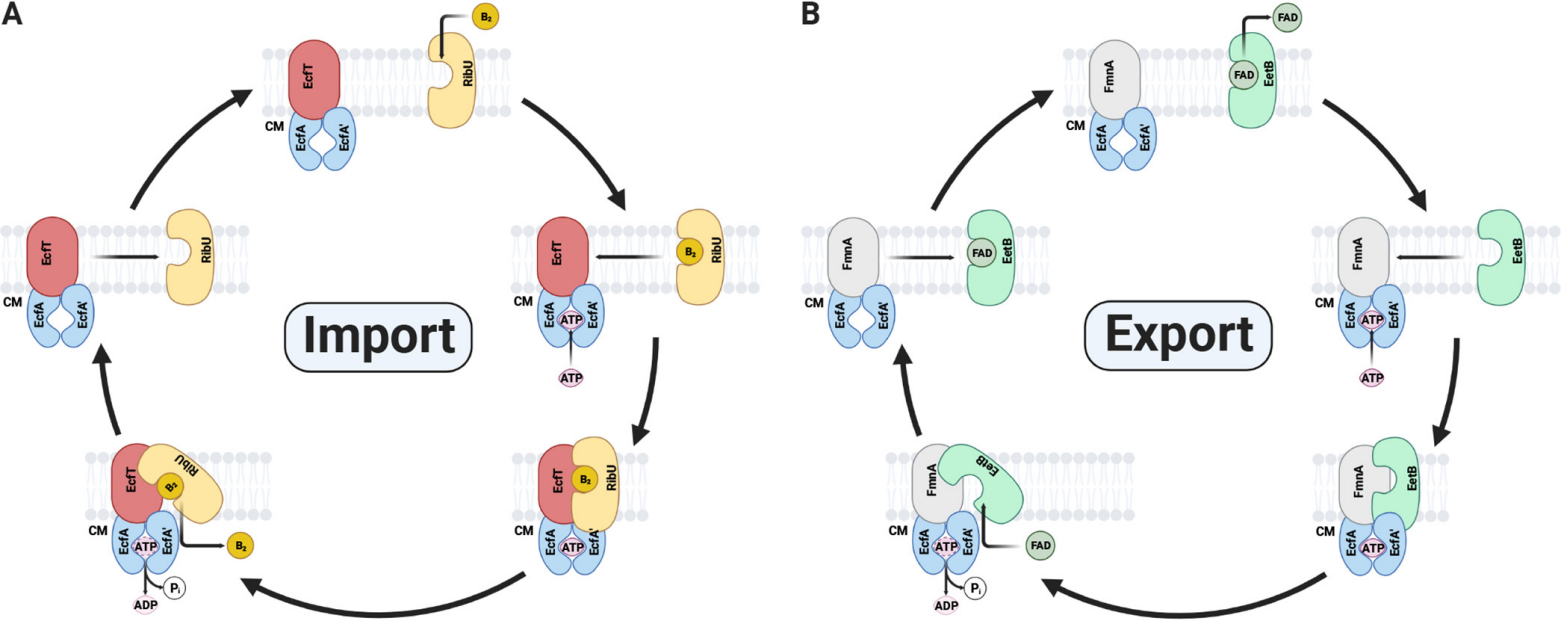
Speculative model of bidirectional ECF transport. (A) Summary of previously proposed models of ECF import. (B) Speculative model of ECF export illustrating how directionality of transport could be achieved through a broadly conserved mechanism. CM refers to cytoplasmic membrane.

While little is known about the mechanism of ECF export, previous research has generated considerable evidence about the basis of ECF import. These studies reveal that ECF-S remains monomeric in the absence of a substrate, likely adopting an “outward”-facing orientation that enables substrate binding from the extracytosolic space (23, 35, 36). Once the substrate is bound, ECF-S undergoes a “toppling” conformational change to an “inward” orientation where it engages the ECF-T–ECF-A–ECF-A′ complex in a manner that facilitates substrate release into the cytosol. ATP hydrolysis in the ECF complex then causes the release of apo-ECF-S, which reverts to its monomeric outward orientation (Fig. 5A) (23, 36). Considering the similarity of subunits, the proposed mechanism of ECF import has implications for how EetB might function in FAD export. Simply reversing the relationship of the ECF-S ligand-binding and transport functions could reverse the direction of transport. While additional structural and biochemical studies will be necessary to definitively address transport mechanisms, ECF export may differ from ECF import in inverting the ECF complex’s unliganded versus substrate-bound ECF-S affinity and the kinetics of ATP hydrolysis/ECF-S release (Fig. 5B).

An interesting aspect of *eetB* and other flavin exporter genes identified in our bioinformatic analyses is their frequent colocalization with an *apbE* gene. This distinguishes the ECF transporter from a previously identified flavin exporter that similarly traffics flavins in some Gram-negative bacteria (2, 3). The association between *eetB* and *apbE* suggests that EetB may be adapted for efficient flavin delivery to ApbE. Indeed, a regulatory mechanism that enables targeted delivery to ApbE might explain why, despite several efforts, we were unable to detect differences in the levels of extracellular flavins within *eetB*-deficient strains. Flavin delivery to ApbE may thus provide an attractive model for future investigations into the mechanism of targeted extracytosolic cofactor delivery in bacteria.

## MATERIALS AND METHODS

### Bacterial strains and culture.

All strains of L. monocytogenes used in this study (Table 1) were derived from the wild-type 10403S strain and cultured in a previously described chemically defined synthetic medium containing 200 μg/mL of streptomycin (37, 38). The deletion of genes was done using allelic exchange with the temperature-sensitive plasmid pKSV7, as previously described (39). The generation of the strains expressing the *ribDEAHT* operon from the pHyper constitutive promoter (pHyper *ribDEAHT* construct) was done by amplifying the *ribDEAHT* operon from B. subtilis and cloning it into the site-specific pPL2 integrating vector. The plasmids were then introduced into L. monocytogenes by conjugation, as previously described (40). Broth growth curves were performed with L. monocytogenes strains from cultures grown overnight in chemically defined synthetic medium at 37°C with shaking (200 rpm). Growth was measured by the optical density at a wavelength of 600 nm (OD_600_), and the growth curves were started at an OD_600_ of 0.03.

**TABLE 1:** Bacterial strains used in this study

Strain	Description	Reference
Listeria monocytogenes		
10403S	Wild type	37
DP-L7376	Δ*ribU*	25
DP-L7197	Δ*eetA*	This study
DP-L7198	Δ*eetB*	This study
DP-L7194	Δ*fmnA*	This study
DP-L7195	Δ*fmnB*	10
DP-L7486	Δ*ecfA*/Δ*ecfA'*	This study
DP-L7487	Δ*ecfT*	This study
DP-L7488	Δ*ecfT*/Δ*fmnA*::Tn	This study
DP-L7489	Δ*ribU*/Δ*eetB*::Tn	This study
DP-L7490	Wild type/pPL2-pHyper *ribDEAHT*	This study
DP-L7491	Δ*ribU*/pPL2-pHyper *ribDEAHT*	This study
DP-L7492	Δ*eetA*/pPL2-pHyper *ribDEAHT*	This study
DP-L7493	Δ*eetB*/pPL2-pHyper *ribDEAHT*	This study
DP-L7494	Δ*fmnA*/pPL2-pHyper *ribDEAHT*	This study
DP-L7495	Δ*fmnB*/pPL2-pHyper *ribDEAHT*	This study
DP-L7496	Δ*ecfA*/Δ*ecfA'*/pPL2-pHyper *ribDEAHT*	This study
DP-L7497	Δ*ecfT*/pPL2-pHyper *ribDEAHT*	This study
Escherichia coli		
DP-E7498	SM10/pPL2-pHyper *ribDEAHT*	This study
DP-E7499	SM10/pKSV7-oriT *eetA*	This study
DP-E7500	SM10/pKSV7-oriT *eetB*	This study
DP-E7501	SM10/pKSV7-oriT *fmnA*	This study
DP-E7502	SM10/pKSV7-oriT *ecfA/ecfA'*	This study
DP-E7503	SM10/pKSV7-oriT *ecfT*	This study
SL-457	Rosetta/pMCSG53 *ribU*	This study
SL-458	Rosetta/pMCSG53 *eetB*	This study

### Ferric iron reductase activity assays.

Strains were grown to mid-exponential phase (OD_600_ = ~0.4 to 0.6) in chemically defined synthetic medium or chemically defined synthetic medium supplemented with 0.5 mM flavin adenine dinucleotide (FAD) and then washed twice with 1× phosphate-buffered saline (PBS). Washed bacteria were resuspended in chemically defined synthetic medium supplemented with 4 mM Ferrozine and normalized to an OD_600_ of 0.5. To conduct the assay, 100 μL of resuspended bacteria was mixed with 100 μL of chemically defined synthetic medium supplemented with 100 mM ferric ammonium citrate and transferred into a 96-well plate in triplicate. Measurements were done using a plate reader with the temperature set at 37°C, and the absorbance was read at 560 nm every 30 s for 1.5 h. Maximal rates (typically over 2 min) were calculated and reported as a percentage of the ferric iron reductase activity of the wild type.

### Recombinant *ribU* and *eetB* expression and FAD pulldown.

The L. monocytogenes 10403S *eetB* and *ribU* genes were cloned into the pMCSG53 vector. The resulting constructs were transformed into E. coli BL21 cells. Cultures of E. coli BL21 containing pMCSG53::*empty*, pMCSG53::*eetB*, and pMCSG53::*ribU* grown overnight were diluted in 5 mL of Luria-Bertani (LB) broth at a final OD_600_ of 0.05. When cell growth reached exponential phase, FAD was added to a final concentration of 1 μM, and gene expression was induced with isopropyl β-d-1-thiogalactopyranoside (IPTG) at a final concentration of 1 mM. Induced cultures were grown overnight at 30°C. Cultures were normalized to an OD_600_ of 1.0, and 1 mL of cells was then collected by centrifugation at 22,100 × *g* for 1 min. Cell pellets were washed twice with 1 mL of deionized water (diH_2_O) and resuspended in 190 μL of diH_2_O to remove free FAD. To facilitate the release of protein-bound FAD, cell suspensions were incubated at 100°C for 20 min and centrifuged at 22,100 × *g* for 1 min. The supernatants were collected for analysis of the flavin content.

### Liquid chromatography-mass spectrometry (MS) for the detection of flavins.

Samples were incubated at −80°C for at least 1 h or up to overnight. The extraction solvent (4 volumes of 100% methanol spiked with internal standards and stored at −80°C) was added to the liquid sample (1 volume) in a microcentrifuge tube. The tubes were then centrifuged at −10°C at 20,000 × *g* for 15 min, and the supernatant was used for subsequent metabolomic analyses. Samples were dried completely using a Genevac EZ-2 Elite system. Samples were resuspended in 50 μL of 50:50 water-methanol and added to an Eppendorf thermomixer at 4°C at 1,000 rpm for 15 min to resuspend the analytes. Samples were then centrifuged at 4°C at 20,000 × *g* for 15 min to remove insoluble debris, and 40 μL of the supernatant was transferred to a 96-deep-well plate (catalog number 5065-4402; Agilent). Samples were analyzed on an Agilent 1290 infinity II liquid chromatography (LC) system coupled to an Agilent 6470 triple-quadrupole mass spectrometer, operating in positive mode, equipped with an Agilent Jet Stream electrospray ionization (ESI) source (LC-ESI-QQQ). Each sample (2 μL) was injected into an Acquity ultraperformance liquid chromatography (UPLC) HSS PFP column (1.8 μm, 2.1 by 100 mm) (catalog number 186005967; Waters) equipped with an Acquity UPLC HSS PFP VanGuard precolumn (100 Å, 1.8 μm, 2.1 mm by 5 mm) (catalog number 186005974; Waters) at 45°C. Mobile phase A was 0.35% formic acid in water, and mobile phase B was 0.35% formic acid in 95:5 acetonitrile-water. The flow rate was set to 0.5 mL/min starting at 0% mobile phase B held constant for 3 min, linearly increased to 50% over 5 min, then linearly increased to 95% B over 1 min, and held at 100% B for the next 3 min. Mobile phase B was then brought back down to 0% over 0.5 min and held at 0% for reequilibration for 2.5 min. The QQQ electrospray conditions were set with a capillary voltage of 4 kV and a nozzle voltage of 500 V, and dynamic multiple-reaction monitoring (MRM) was used with a cycle time of 500 ms. Transitions were monitored in positive mode for two analytes, riboflavin and FAD. The transitions for riboflavin and FAD were 377.1 *m/z* to 243 *m/z* and 786.1 *m/z* to 348 *m/z*, respectively. Authentic standards were purchased for riboflavin (riboflavin B_2_; Supelco) and FAD (flavin adenine dinucleotide disodium salt hydrate; Sigma-Aldrich) to make 1-mg/mL stock solutions in methanol. These solutions were used to prepare a 10-point calibration curve, ranging from 3.1 nM to 0.2 mM for riboflavin and from 3.9 nM to 1 mM for FAD. Data analysis was performed using MassHunter Quant software (version B.10; Agilent Technologies) and confirmed by comparison with standards. Normalized peak areas were calculated by dividing the raw peak areas of the targeted analytes by the averaged raw peak areas of two internal standards (melatonin and kynurenic acid).

### Structural modeling of transporter subunits.

The AlphaFold model of EetB (accession number AF-Q927J9-F1) was downloaded from the UniProt database (41). RibU-EcfT-EcfA-EcfA′ and EetB-FmnA-EcfA-EcfA′ complex models were generated with AlphaFold-multimer, an AlphaFold model trained on protein complexes, using default settings on the ColabFold platform (41–43). The AlphaFold model quality was evaluated on the basis of predicted local distance difference test (pLDDT) and predicted aligned-error (PAE) scores (41–44). Structure root mean square deviation values were calculated using the align command in PyMOL v2.5.1 (http://www.pymol.org/pymol).

### Liquid chromatography-mass spectrometry analysis of trypsin-digested proteins.

Samples of trypsin-digested proteins were analyzed using a Synapt G2-Si ion mobility mass spectrometer that was equipped with a nanoelectrospray ionization source (Waters, Milford, MA). The mass spectrometer was connected in line with an Acquity M-class ultraperformance liquid chromatography system that was equipped with trapping (Symmetry C_18_ [inner diameter, 180 μm; length, 20 mm; particle size, 5 μm]) and analytical (HSS T3 [inner diameter, 75 μm; length, 250 mm; particle size, 1.8 μm]) columns (Waters). Data-independent, ion mobility-enabled, high-definition mass spectra and tandem mass spectra were acquired in the positive-ion mode (45–48). Data acquisition was controlled using MassLynx software (version 4.1), and tryptic peptide identification and relative quantification using a label-free approach were performed using Progenesis QI for Proteomics software (version 4.0; Waters) (49–51). Data were searched against the Listeria monocytogenes serotype 1/2a (strain 10403S) protein database to identify tryptic peptides, with carbamidomethylcysteine as a fixed posttranslational modification and methionine sulfoxide and threonine flavinylation as variable posttranslational modifications (52). Calculation of the percentage of flavinylation for each bacterial strain was performed by dividing the abundance of a residue/peptide bearing a flavinylation modification by the total abundance and multiplying this value by 100.

### Genome collection for analysis of *eetB* gene clusters.

The 30,238 bacterial and 1,672 archaeal genomes from the Genome Taxonomy Database (GTDB), release 05-RS95 (17 July 2020), were downloaded with the taxonomy and predicted protein sequences (33, 34).

### Functional annotation of *eetB* gene clusters.

Protein sequences were functionally annotated based on the accession number of their best Hmmsearch, version 3.3 (E value cutoff of 0.001), match against the KOfam database (downloaded on 18 February 2020) (53, 54). Domains were predicted using the same Hmmsearch procedure against the Pfam database, version 33.0 (55). SIGNALP, version 5.0, was run to predict the putative cellular localization of the proteins using the parameters -org arch in archaeal genomes and -org gram+ in bacterial genomes (56). Prediction of transmembrane helices in proteins was performed using TMHMM, version 2.0 (default parameters) (57).

### Detection of *eetB* gene clusters and association with flavinylated systems.

The five genes downstream and upstream of *eetB* (Pfam family PF07456) genes were first collected. The *eetB* gene clusters were then assigned to flavinylated systems based on the presence of previously reported key genes (4).
